# Does early pregnancy exposure to macrolide antibiotics lead to major birth defects? A systematic review and meta-analysis

**DOI:** 10.3389/fpubh.2026.1513736

**Published:** 2026-01-16

**Authors:** Jing Zhang, Quanhong Li, Yanbin Yang, Hong Su, Yu Sun, Xian Huang, Ying Song, Yangping Zhang

**Affiliations:** 1 Department of Obstetrics, Kunming City Maternal and Child Health Hospital, Kunming, China; 2 Department of Pharmacy, Kunming Children's Hospital, Kunming, China; 3 Rehabilitation Department, Kunming Children's Hospital, Kunming, China

**Keywords:** early pregnancy, macrolide antibiotics, major birth defects, meta-analysis, systematic review

## Abstract

**Background:**

Macrolide antibiotics are frequently used to treat infections in women during early pregnancy. However, the association between early pregnancy exposure to macrolide antibiotics and major birth defects remains uncertain. Clarifying this risk is crucial for public health and clinical decision-making.

**Methods:**

This study systematically searched five databases—Web of Science, PubMed, Embase, Cochrane, and Scopus—for studies exploring the relationship between early pregnancy exposure to macrolide antibiotics and major birth defects. Two independent researchers conducted the literature screening, data extraction, and bias risk assessment. Meta-analysis was performed using the metan command in STATA 16 to pool the data.

**Results:**

Nine studies were included, selected from 6,002 articles, comprising seven cohort studies and two case-control studies. These studies covered 40,218 patients exposed to macrolide antibiotics in early pregnancy. Due to low heterogeneity among studies, a fixed-effects model was used for the meta-analysis. The results showed no significant association between early pregnancy exposure to macrolide antibiotics and the risk of major birth defects (OR = 1.05, 95% CI [0.97, 1.13]). Subgroup analysis of 11 types of major birth defects also found no such association, particularly for birth defects of the heart (OR = 1.09, 95% CI [0.96, 1.23]). Low heterogeneity and reduced risk of bias further enhanced the reliability of the findings.

**Conclusion:**

Existing evidence suggests that early pregnancy use of macrolide antibiotics does not increase the risk of major birth defects. In clinical practice, healthcare providers and pregnant women can use these antibiotics cautiously based on the specific infection situation.

## 1 Introduction

Antibiotics are among the most commonly prescribed medications during pregnancy worldwide, accounting for approximately 80% of all prescriptions during this period, with around 20–25% of women receiving antibiotic treatment during pregnancy (1). Among these, macrolide antibiotics have become widely used for the treatment of various infectious diseases due to their broad spectrum of indications and good tolerability. Macrolides are commonly prescribed for the treatment of upper and lower respiratory tract infections, urinary tract infections, skin and soft tissue infections, as well as peptic ulcers caused by *Helicobacter pylori* infection (2). Additionally, macrolides are an important alternative for patients allergic to penicillin, especially during pregnancy. For instance, azithromycin, a cost-effective and broad-spectrum macrolide, is widely used in the prevention and treatment of perinatal and neonatal infections due to its long half-life in placental tissue, which allows it to maintain a high drug concentration (3). Specific applications include the treatment of sexually transmitted diseases (4), intermittent malaria prophylaxis (5), and the prevention of post-cesarean section infections (6). Despite the widespread clinical use of macrolides, their safety during pregnancy, particularly regarding fetal development, remains controversial.

Although macrolides are extensively used in clinical practice, their safety during pregnancy, especially concerning fetal development, remains a subject of debate. Previous studies have suggested that the use of macrolides during pregnancy may be associated with adverse outcomes in offspring, including cerebral palsy, epilepsy, asthma, obesity, and neurological dysfunction (7). One large cohort study indicated that compared to penicillin, the use of macrolide antibiotics during pregnancy significantly increased the risk of major birth defects, particularly cardiovascular malformations (8). A population-based study from the Swedish Medical Birth Registry, conducted between 1995 and 2002, further found a significant association between maternal erythromycin use during early pregnancy and an increased risk of congenital cardiovascular defects in offspring (9). However, another nationwide cohort study did not find any association between macrolide use during pregnancy and an increased risk of major birth defects (10). To address these controversies, Keskin-Arslan et al. (11) conducted a meta-analysis on the relationship between macrolide use during pregnancy and birth defects. The study summarized several observational studies and concluded that the risk of major congenital malformations and congenital heart defects did not significantly increase after the use of any macrolide antibiotics during the first trimester of pregnancy. Although the study by Keskin-Arslan et al. (11) provided preliminary analysis on this issue, the differences between studies—such as the diversity of research methods, sample heterogeneity, and variations in study periods—mean that no consistent conclusions have been reached regarding the relationship between macrolide use during pregnancy and the risk of birth defects.

Given that the early stages of pregnancy are a critical period for fetal development and are particularly sensitive to drug exposure, the aim of this study is to further investigate the relationship between exposure to macrolide antibiotics during early pregnancy and the risk of major birth defects. By synthesizing existing literature and data, we hope to provide clearer evidence that will assist clinicians and pregnant women in making more scientifically informed decisions regarding antibiotic use during pregnancy.

## 2 Methods

### 2.1 Research protocol

This study follows the Preferred Reporting Items for Systematic Reviews and Meta-analyses (PRISMA) guidelines (12). The research protocol has been pre-registered on the PROSPERO website with the registration number CRD42023390012 to ensure transparency and adherence to the study plan.

### 2.2 Inclusion/exclusion criteria

We developed strict inclusion and exclusion criteria based on the PICOS framework. The specific criteria are as follows:

Inclusion criteria: 1. Population (P): Pregnant women, particularly during early pregnancy (before 12 weeks of gestation). 2. Intervention (I): Exposure to macrolide antibiotics during early pregnancy, including but not limited to erythromycin, clarithromycin, and azithromycin. 3. Comparison (C): The control group consists of pregnant women who were not exposed to macrolide antibiotics or were exposed to other types of antibiotics but not to macrolides. 4. Outcomes (O): Studies reporting major birth defects will be included, including but not limited to congenital heart defects, neural tube defects, cleft lip and palate, congenital limb malformations, congenital hearing loss, and other major developmental anomalies. 5. Study design (S): Includes observational studies (e.g., cohort studies, case-control studies), with priority given to studies that provide sufficient data to calculate effect sizes.

Exclusion criteria: 1. Studies focusing on non-pregnant women. 2. Studies where exposure to macrolide antibiotics occurred after early pregnancy (i.e., after 12 weeks of gestation). 3. Studies where participants were exposed to non-macrolide antibiotics or used other antibiotics concurrently within the same pregnancy. 4. Studies that did not report any major birth defects or did not provide relevant outcome data. 5. Reviews, case reports, conference abstracts, letters, animal studies, and other research not applicable to human population analysis.

### 2.3 Literature search

To obtain the most comprehensive collection of relevant studies, we employed a systematic search strategy. The search terms included (macrolide or macrolides or erythromycin or clarithromycin or azithromycin) and (pregnant or pregnancy or pregnancies or maternal or prenatal or gestation or gestational). Databases searched included Web of Science, PubMed, Embase, Cochrane Library, Scopus, and Reprotox. The search time range covered all studies from the establishment of these databases to August 4, 2024. In addition, we manually searched the reference lists of all relevant systematic reviews and meta-analyses to identify additional studies that may meet the inclusion criteria. A detailed search strategy is available in Supplementary Table S1.

### 2.4 Literature screening, data extraction, and risk of bias assessment

Literature screening, data extraction, and risk of bias assessment were independently performed by two researchers. Before screening, duplicate records were removed using EndNote X9.1 software. The two researchers independently reviewed the titles and abstracts of the studies, initially excluding those that clearly did not meet the inclusion criteria. After obtaining the full texts of the studies passing the initial screening, both researchers independently assessed whether the studies met the inclusion criteria. We used a pre-designed data extraction form to collect relevant information. For the risk of bias assessment, the ROBINS-I (Risk of Bias in Non-randomized Studies of Interventions) tool was employed. Any disagreements between the two researchers were first resolved through discussion (Jing Zhang and Quanhong Li). If consensus could not be reached, a senior researcher (Ying Song) was consulted for arbitration, and the final decision was made based on their judgment.

The extracted information included: 1. Basic information: Authors, publication year, country, study type, patient source, sample size, maternal age at the time of pregnancy, type of macrolide antibiotics, and the timing of drug initiation. 2. Outcome indicators: Any major birth defects. 3. Key information for risk of bias assessment.

The risk of bias in cohort and case-control studies was assessed using the ROBINS-I tool. The risk of bias was evaluated across seven domains: Bias due to confounding, bias due to selection of participants, bias due to exposure assessment, bias due to misclassification during follow-up, bias due to missing data, bias due to measurement of the outcome, and bias due to selective reporting of results. The overall risk of bias for each study was determined.

### 2.5 Statistical analysis

All statistical analyses will be conducted using STATA 16 software. The combined effect size will be expressed as odds ratios (OR) and their 95% confidence intervals (CI). When studies report relative risk (RR), we will convert it to OR for data pooling in the meta-analysis. Specifically, we will use the following formula for conversion: OR = RR × [1 - P0]/[P0 × (RR - 1)], where P0 represents the population incidence of the disease (13). To ensure the accuracy of OR conversion when the event rate is low, we will apply a correction strategy for rare events. This involves using Poisson regression-based rare event correction methods to adjust the OR estimates and reduce bias due to low event rates. Additionally, we will conduct a sensitivity analysis comparing the traditional RR to OR conversion formula with the corrected OR estimates to ensure the robustness and reliability of the results. To further control the impact of low event rates on the study’s conclusions, we will perform subgroup analysis for studies with extremely /low event rates and separate them from studies with higher event rates, improving the stability and accuracy of the meta-analysis results. These methods will ensure that the study results remain reliable even in the presence of low event rates. We will assess heterogeneity between studies using Cochran’s *Q* test and *I*^2^ statistics. If the *p*-value > 0.05 and *I*^2^ ≤ 50%, we will consider the heterogeneity between studies to be low and use a fixed-effect model for the meta-analysis. Otherwise, significant heterogeneity will be present, and we will further explore its sources and use a random-effects model for analysis. Additionally, we will conduct subgroup analysis according to the different subtypes of major birth defects. A funnel plot will be created to detect publication bias, and further quantitative assessment of publication bias will be performed using the Egger and Begg tests.

## 3 Results

### 3.1 Article searching results

**Figure 1:**
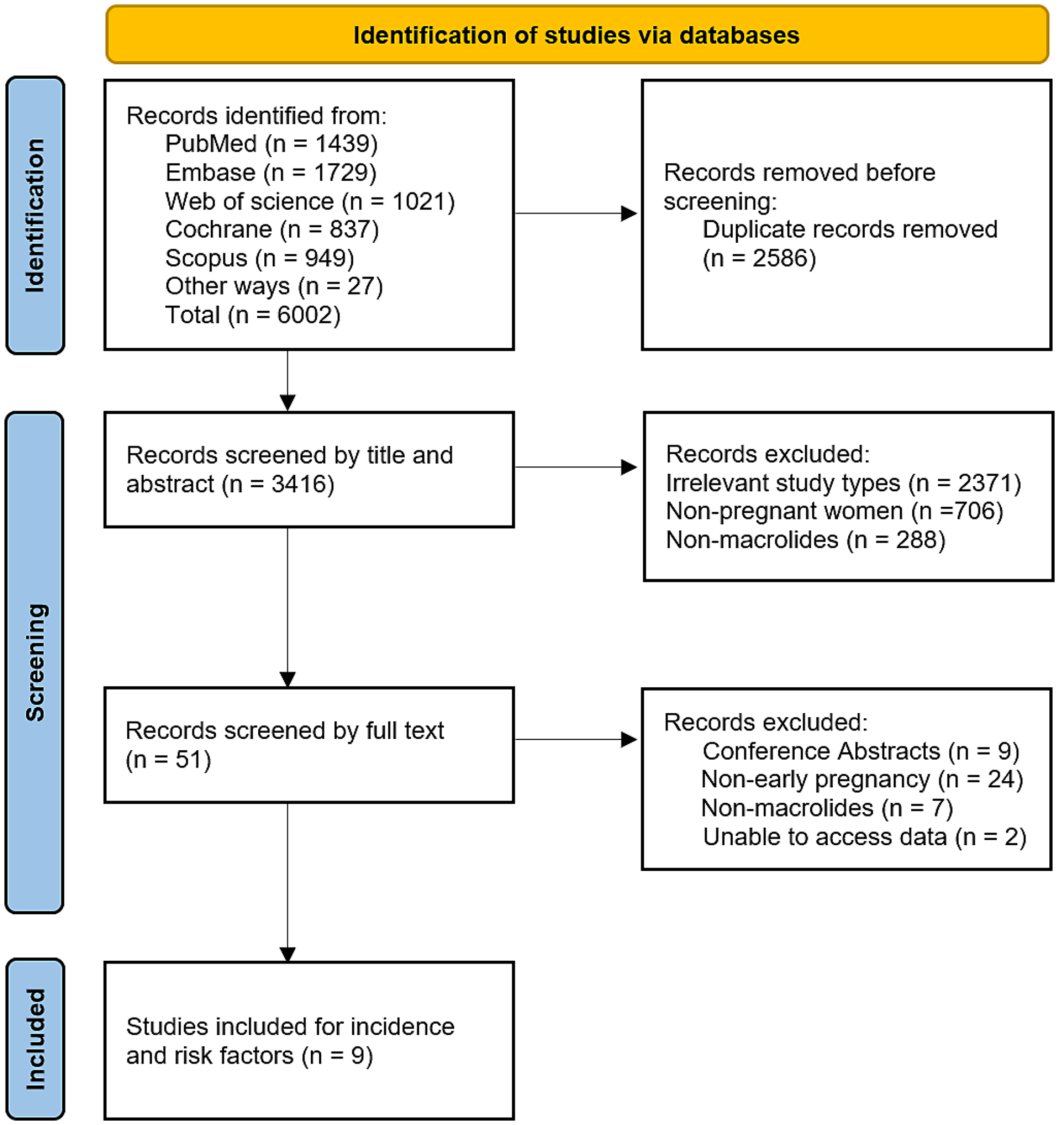
PRISMA flow chart.

A total of 5,975 articles were identified from the five databases mentioned above, and an additional 27 articles were retrieved from reference lists and reviews, resulting in a total of 6,002 articles. After removing 2,586 duplicate records, the remaining 3,416 articles were screened based on titles and abstracts. Following this, 3,365 studies were excluded due to non-relevant study types, non-pregnant women, or the use of non-macrolide antibiotics. Full-text review of the remaining 51 articles led to the exclusion of conference abstracts, studies on non-early pregnancy, studies with no exposure to macrolides, and studies with unavailable data. Ultimately, nine studies were included in the final analysis (8, 10, 14–20). The article selection process is shown in Figure 1.

### 3.2 Basic information and results of the risk of bias assessment

The nine included studies consisted of seven cohort studies (8, 10, 14, 17–20) and two case-control studies (15, 16). The studies were published between 1999 and 2021, and the locations of the studies included Denmark (10, 20), Israel (18, 19), Norway (14), England (15), Canada (17), Hungary (16), the UK (8). A total of 40,218 pregnant women were exposed to macrolide antibiotics, and 1,089 children were born with birth defects. The sample sizes of the studies ranged from 113 to 13,019. Four studies reported the average age of pregnant women, which ranged from 25.5 to 30.5 years. In all studies, the pregnant women were exposed to various macrolide antibiotics (including azithromycin, clarithromycin, erythromycin, roxithromycin, and spiramycin) during the first trimester of pregnancy.

**Figure 2:**
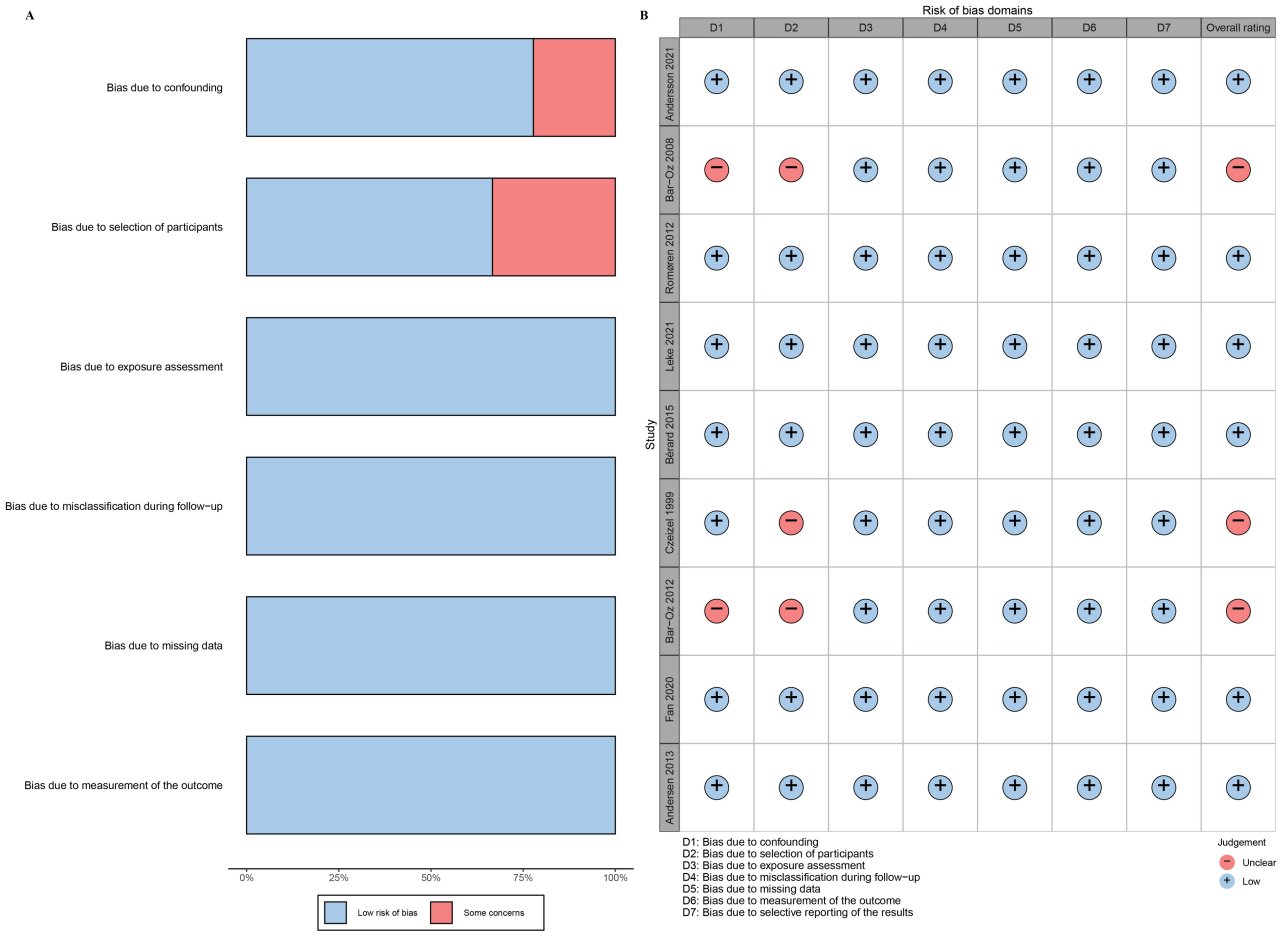
Results of the risk of bias assessment. **(A)** Assessment results for each item; **(B)** Evaluation results for individual studies.

Of the nine included studies, six had a low risk of bias, while three had a moderate risk of bias. The primary sources of bias were participant selection and confounding, which contributed to the overall risk of bias in the studies. The results of the risk of bias assessment are shown in Figure 2.

### 3.3 Meta-analysis results

#### 3.3.1 Any major birth defect

**Figure 3:**
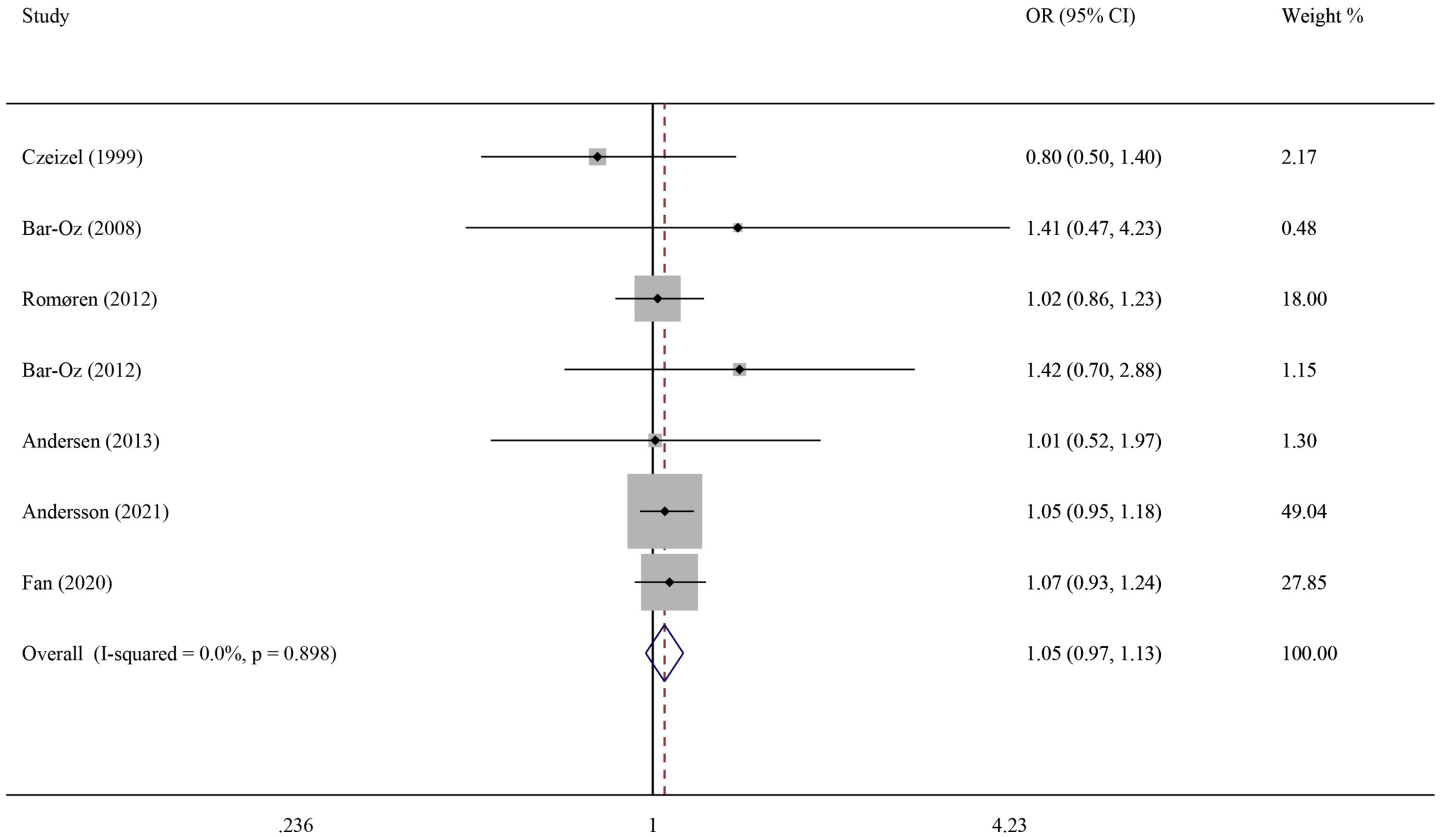
Meta-analysis results for any major birth defect.

Seven studies reported on the relationship between early pregnancy exposure to macrolide antibiotics and any major birth defects. As there was no significant heterogeneity between the studies (*I*^2^ = 0, *p* > 0.05), a fixed-effect model was used for the meta-analysis. The results showed that early pregnancy exposure to macrolide antibiotics did not increase the risk of any major birth defect (OR = 1.05, 95% CI [0.97, 1.13]) (Figure 3).

#### 3.3.2 Subtypes of major birth defects

**Figure 4:**
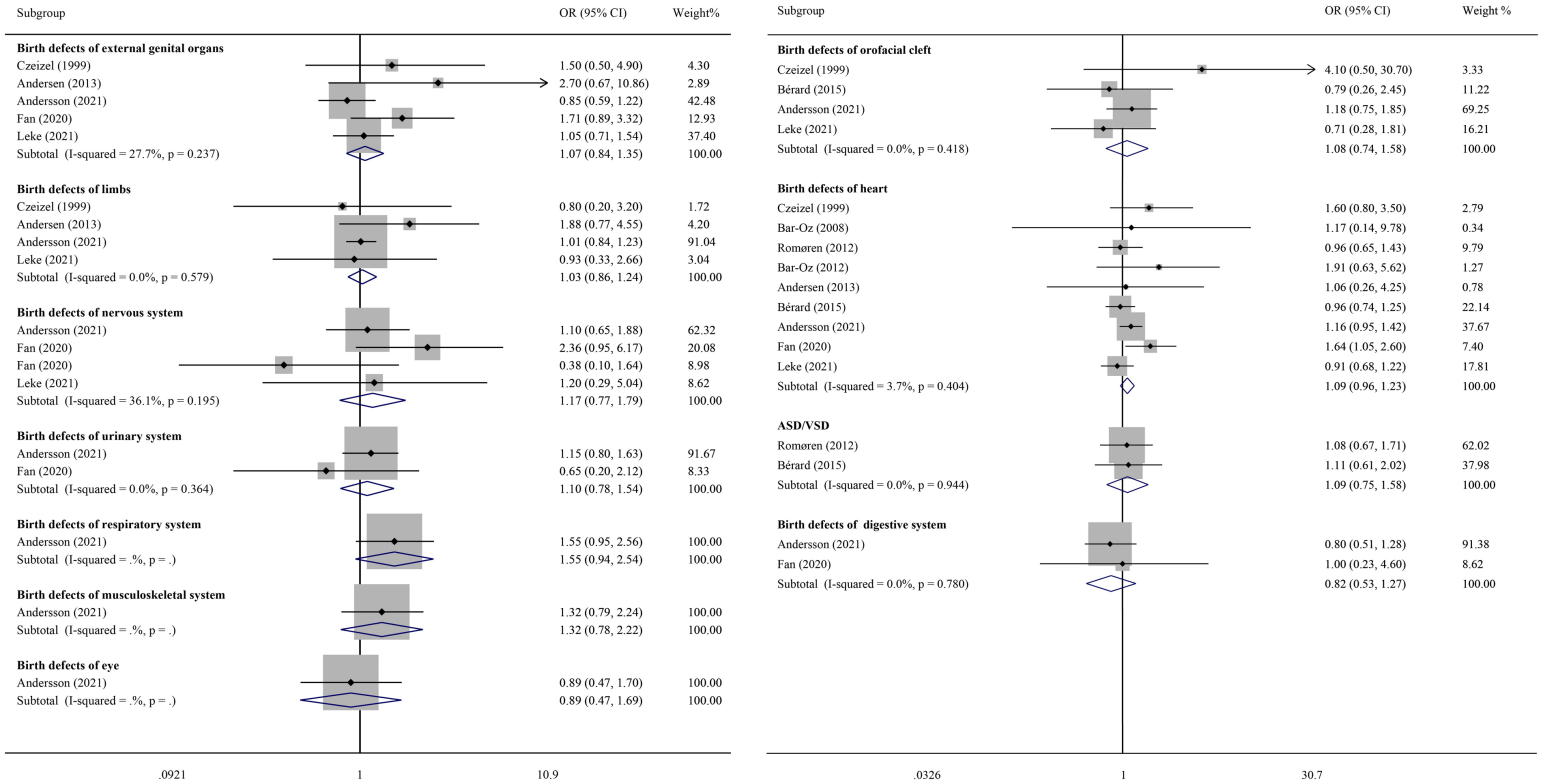
Subtypes of major birth defects.

The nine included studies reported on 11 subtypes of major birth defects. Birth defects of the respiratory system, musculoskeletal system, and eye were only reported in the study by Andersson et al. (10). The results showed that early pregnancy exposure to macrolide antibiotics did not increase the risk of these three types of major birth defects (Figure 4).

**Figure 5:**
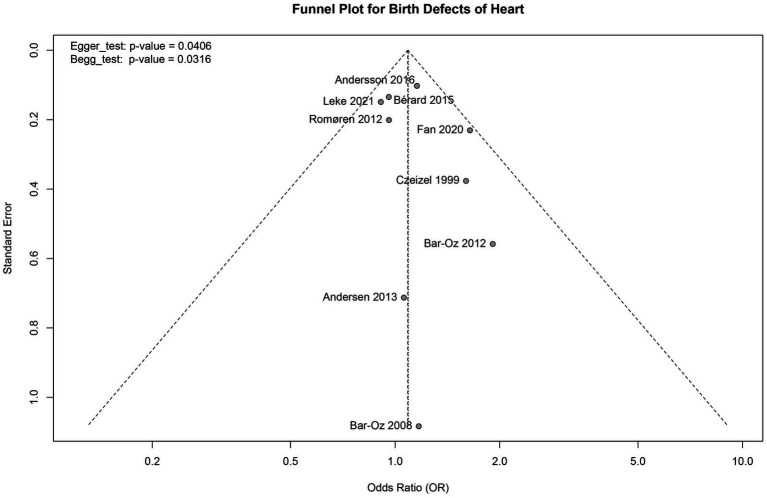
Funnel plot.

Eight types of major birth defects were reported in two or more studies, including defects of the external genital organs, limbs, nervous system, urinary system, orofacial clefts, heart (ASD/VSD), and the digestive system. Since *I*^2^ was less than 50% and *p* > 0.05, a fixed-effect model was used for the meta-analysis. The results showed that early pregnancy exposure to macrolide antibiotics did not increase the risk of these eight major birth defects (Figure 5). Additionally, a random-effects model was used for the meta-analysis, and the results also indicated that early pregnancy exposure to macrolides did not increase the risk of these eight birth defects (Supplementary Figure S1).

### 3.4 Publication bias

All studies reported a relationship between early pregnancy exposure to macrolide antibiotics and heart birth defects. Therefore, a funnel plot was constructed based on heart birth defects as the outcome measure. The asymmetry of the funnel plot suggests the potential for publication bias (Figure 5). Further analysis using the Egger test and Begg test both yielded *p*-values < 0.05, indicating the likelihood of publication bias.

## 4 Discussion

The use of macrolide antibiotics during pregnancy for the treatment or prevention of infections is a widespread clinical practice globally. However, there remains considerable debate over whether their use may adversely impact pregnancy outcomes. Pharmacokinetics and pharmacodynamics during pregnancy differ from those in non-pregnant states, which may affect the action of the drug on both the mother and the fetus. Antibiotic use could potentially impact the embryo indirectly by altering maternal endocrine and metabolic functions or directly through the placental barrier or breast milk, thereby affecting the fetus or neonate (21). Macrolide antibiotics, as a class of broad-spectrum antibiotics, are favored in the treatment of infectious diseases during pregnancy, particularly for patients who are allergic to penicillin, where they are often used as an alternative (22). Currently, nearly 50% of pregnancies are unplanned, and 5–10% of women of reproductive age may become pregnant at any given time (23). This implies that some women may use antibiotics on their own or based on a doctor’s prescription without realizing they are pregnant. The critical period for fetal malformations is considered to be from the 3rd to the 8th week of pregnancy (24).

To date, the primary concern regarding the use of macrolide antibiotics during pregnancy has been whether they increase the risk of cardiac malformations. Some studies suggest that macrolide drugs may affect the electrophysiological activity of the embryonic heart, leading to bradycardia or arrhythmias, which could impact blood flow and potentially cause hypoxia, thereby resulting in cardiac malformations (25). However, the comprehensive analysis based on data from 9 observational studies in this research found no significant association between early pregnancy exposure to macrolides and cardiac birth defects or atrial/ventricular septal defects. Differences in data collection methods, statistical analyses, sample sizes, and control of confounding factors across studies may account for the inconsistencies in results. For example, in Herrett et al.’s (26) study, birth defect information was collected from general practitioners’ historical records when the child was 3 years old, whereas Andersson et al.’s (10) study obtained data from inpatient and outpatient care records. Early studies may be influenced by bias, small sample sizes, or inadequate control of other confounding factors. In contrast, this comprehensive analysis integrated data from multiple studies, reducing such issues and drawing a more robust conclusion that early pregnancy exposure to macrolide antibiotics is not significantly associated with fetal heart malformations.

In addition, several studies have reported congenital malformations following maternal exposure to macrolide antibiotics, including cardiac defects (9, 27), urinary system malformations (28), anencephaly, and limb defects (29). However, the summary analysis of major birth defects and their 11 subtypes in this study shows that early pregnancy exposure to macrolide antibiotics does not increase the risk of major birth defects. However, our pooled analysis of major birth defects and their 11 subtypes indicated that early exposure to macrolide antibiotics during pregnancy did not increase the risk of major birth defects. Our findings are consistent with some recently published comprehensive meta-analyses, though there are differences. This result aligns with the meta-analysis by Keskin-Arslan et al. (11), which found no significant increase in the risk of major congenital malformations (OR 1.06 [95% CI 0.99, 1.13]) or congenital heart defects (OR 1.05 [95% CI 0.92, 1.19]) after exposure to macrolide antibiotics during the first trimester of pregnancy. Although our study is similar to that of Keskin-Arslan et al. (11), their study lacked a quantitative analysis of several important study results and relied on descriptive analysis (10, 17). Additionally, Keskin-Arslan et al.’s (11) study primarily focused on the effects of exposure to macrolide antibiotics at any time during pregnancy on pregnancy outcomes, whereas our study specifically concentrated on early pregnancy, offering a more focused approach. This is the main distinction between the two studies, but the similar results provide further evidence of the safety of early exposure to macrolide antibiotics. Similarly, Fan et al. (30) focused primarily on the association with neurological outcomes in children (such as cerebral palsy and epilepsy) and reported an increased risk of miscarriage, but their analysis of malformation outcomes did not involve a strict stratification by early pregnancy. The study by Mallah et al. (31) reported a slight increase in the risk of musculoskeletal and digestive system malformations; however, one major limitation of this study was that its control group consisted of a “mixed population” (those unexposed to any drugs and those exposed to other antibiotics), which could have introduced unmanageable confounding bias. In contrast, our study used a more rigorous definition of the control group, and all exposures were explicitly defined as occurring during early pregnancy, which strengthened the internal validity of the results. Moreover, the study by Omranipoor et al. (32) focused on spontaneous abortion, while our study assessed major birth defects, thus providing evidence of the safety of macrolide antibiotics during pregnancy from a different perspective.

An important issue that requires in-depth exploration is confounding by indication. In this study, macrolide antibiotics were prescribed to pregnant women for the treatment of specific infections, such as respiratory infections, urogenital infections, and sexually transmitted diseases. Therefore, any observed potential risk associations could theoretically stem either from the drug itself or from the underlying infections being treated, or a combination of both. Certain infections, especially severe infections that cause high fever, have been established as independent risk factors for birth defects (33, 34). For instance, untreated urogenital infections have been linked to preterm birth and low birth weight, while certain viral infections are directly associated with specific birth defects (35, 36). In the original studies included in this meta-analysis, the control group typically consisted of pregnant women who were not exposed to macrolide antibiotics, including healthy pregnant women and those who were prescribed alternative antibiotics, such as penicillins, for other infections. This study design helps to some extent in distinguishing between the effects of the drug and the effects of the infection. More importantly, the results from our subgroup analysis show no significant association between macrolide antibiotics and any specific type of birth defect. If the underlying infection were the primary teratogenic factor, we might expect to observe a significantly increased risk of certain birth defects related to specific infections, but such a pattern was not found in this analysis. This finding indirectly supports the notion that the “lack of risk association” is more likely to reflect the relative safety of the drug rather than being solely due to confounding by indication. Nonetheless, due to limited information on the severity of infections and specific pathogens at the individual study level, we were unable to perform quantitative adjustments for these factors in this meta-analysis. Therefore, when interpreting the conclusion of “no significant risk,” clinicians should recognize that for severe infections requiring macrolide antibiotic treatment, the therapeutic benefits are likely to far outweigh any small absolute risks not identified in this study. In contrast, for mild infections, the risks and benefits of all available antibiotics should be carefully weighed.

The strengths of this study are as follows: First, the very low heterogeneity across the included studies ensures consistency in the results. Second, the low risk of bias enhances the reliability of the meta-analysis outcomes. This provides strong evidence supporting the use of macrolide antibiotics. Additionally, this study focused exclusively on the effects of macrolide antibiotics during early pregnancy on major birth defects, making the findings more specific. All nine studies included in this analysis used individual-level data from various national registries, increasing the generalizability of the results and minimizing risks of selection, information bias, and follow-up loss (10). However, there are several limitations to consider. (1) Potential confounding factors and compliance issues: Since all the included studies were observational, we could not fully eliminate potential confounding factors. Variations in maternal compliance with macrolide antibiotics represent an important source of bias. Studies show significant variability in compliance among pregnant women, with adherence to erythromycin ranging from 45 to 100% (37). This variation could result in discrepancies between actual drug exposure and expected exposure, potentially influencing the assessment of birth defect risks. Moreover, pregnant women may have other comorbid conditions (e.g., diabetes, epilepsy) that are known to be associated with certain major birth defects, such as congenital cardiovascular malformations (38). Additionally, pregnant women may be simultaneously exposed to other medications or experience significant differences in socioeconomic status, diet, psychological stress, and lifestyle, all of which could confound the risk of birth defects. While some original studies adjusted for these confounding factors, the depth and methods of adjustment varied, which could affect the accuracy and interpretability of our results. Therefore, future research should more systematically control for these potential confounding factors to improve the accuracy of the results. (2) Data sources and registry issues: All the original studies included in this analysis were observational, relying on data from diverse sources, often dependent on birth defect registries from different countries or regions. However, the quality, completeness, and standardization of these registries may vary, particularly in terms of how birth defects are defined and recorded. These differences could contribute to inconsistencies in results across studies and affect the reliability of our pooled analysis. For instance, some registries may not fully capture all types of birth defects, or there may be information loss or errors during data collection, potentially leading to underestimation or overestimation of the association between macrolide antibiotics and birth defects. Future studies should rely more heavily on standardized birth defect registries and strengthen data quality control to reduce potential registry bias. (3) Language and publication bias: This study only included English-language articles, which may have led to language bias by excluding important studies in other languages. Particularly, research from non-English-speaking countries may have been omitted, potentially offering different contexts and sample characteristics that could influence the generalizability of the results. Moreover, the funnel plot showed some asymmetry, suggesting potential publication bias. Negative results or studies with small sample sizes are often less published or harder to obtain, which could skew our interpretation of the results. Although the majority of the included studies had large sample sizes and high quality, which somewhat mitigates the effect of this bias, caution should still be exercised in interpreting the results. To minimize this bias, future research should consider including multilingual literature and try to include studies with small samples or negative results to enhance the reliability and external validity of the findings.

It is important to note that the primary aim of this study was to provide clearer evidence on the safety of using macrolide antibiotics during pregnancy. By systematically integrating data from 40,218 cases of early pregnancy antibiotic exposure and performing subgroup analyses on 11 types of major birth defects, our findings did not show a statistically significant association between macrolide antibiotic use and an increased risk of birth defects. This finding was also confirmed for high-concern subtypes such as cardiac malformations, suggesting that the existing epidemiological evidence generally supports the relative safety of these drugs. However, we also acknowledge that limitations inherent in observational studies, such as potential confounding, compliance differences, and exposure measurement bias, may still affect the interpretation of results. Therefore, while the current comprehensive evidence does not indicate a marked increase in risk, we cannot assert that macrolide antibiotics are “absolutely safe” during early pregnancy. This study provides important insights into the safety issue, but further high-quality prospective studies are needed to fully validate their safety. Clinically, the use of these drugs should still be weighed against the specific infection, individual maternal characteristics, and risk assessments.

## 5 Conclusion

Current evidence suggests that early pregnancy exposure to macrolide antibiotics is not significantly associated with any major birth defects or their subtypes, including the widely concerned cardiovascular defects. Although the available data do not indicate an increased risk of major birth defects, due to the limitations of the existing studies, clinicians are still advised to exercise caution when selecting macrolide antibiotics. The risks and benefits should be carefully weighed based on the specific infection. Future research may further validate this conclusion and provide more definitive evidence.

## Data Availability

The original contributions presented in the study are included in the article/Supplementary material, further inquiries can be directed to the corresponding authors.
