# Supplementary material for: Does early pregnancy exposure to macrolide antibiotics lead to major birth defects? A systematic review and meta-analysis
Source: Front Public Health. 2026 Jan 16;14:1513736. doi: 10.3389/fpubh.2026.1513736 (PMC12855505; doi:10.3389/fpubh.2026.1513736)
Supplement: Supplementary file 1 [file Supplementary_file_1.docx]

**Does Early Pregnancy Exposure to Macrolide Antibiotics Lead to Major Birth Defects? A Systematic Review and Meta-Analysis**

**Table 1: Article search strategy**

| **1.Web of Science**  (TS=(pregnant or pregnancy or pregnancies or maternal or prenatal or gestation or gestational)) AND TS=(macrolide or macrolides or erythromycin or clarithromycin or azithromycin)  **2.PubMed**  #1: "macrolide"[Title/Abstract] OR "macrolides"[Title/Abstract] OR "erythromycin"[Title/Abstract] OR "clarithromycin"[Title/Abstract] OR "azithromycin"[Title/Abstract]  #2: "Macrolides"[MeSH Terms] OR "Erythromycin"[MeSH Terms] OR "Clarithromycin"[MeSH Terms] OR "Azithromycin"[MeSH Terms]  #3: #1 OR #2 146,469  #4: "pregnant"[Title/Abstract] OR "pregnancy"[Title/Abstract] OR "pregnancies"[Title/Abstract] OR "maternal"[Title/Abstract] OR "prenatal"[Title/Abstract] OR "gestation"[Title/Abstract] OR "gestational"[Title/Abstract]  #5: "Pregnant Women"[MeSH Terms] OR "Pregnancy"[MeSH Terms]  #6: #4 OR #5  #7: #3 AND #6  **3. Embase**  #1: macrolide:ab,ti OR macrolides:ab,ti OR erythromycin:ab,ti OR clarithromycin:ab,ti OR azithromycin:ab,ti  #2: 'macrolide'/exp  #3: 'macrolides'/exp  #4: 'erythromycin'/exp  #5: 'clarithromycin'/exp  #6: 'azithromycin'/exp  #7: #1 OR #2 OR #3 OR #4 OR #5 OR #6  #8: pregnant:ab,ti OR pregnancy:ab,ti OR pregnancies:ab,ti OR maternal:ab,ti OR prenatal:ab,ti OR gestation:ab,ti OR gestational:ab,ti  #9: 'pregnancy'/exp  #10: 'maternal'/exp  #11: 'prenatal'/exp  #12: 'gestation'/exp  #13: #8 OR #9 OR #10 OR #11 OR #12  #14: #7 AND #13  #15: #7 AND #13 AND ([article]/lim OR [article in press]/lim OR [review]/lim) AND [english]/lim AND [clinical study]/lim  **4.Cochrane**  #1: (macrolide or macrolides or erythromycin or clarithromycin or azithromycin):ti,ab,kw  #2: MeSH descriptor: [Macrolides] explode all trees  #3: MeSH descriptor: [Erythromycin] explode all trees  #4: MeSH descriptor: [Clarithromycin] explode all trees  #5: MeSH descriptor: [Azithromycin] explode all trees  #6: #1 or #2 or #3 or #4 or #5  #7: (pregnant or pregnancy or pregnancies or maternal or prenatal or gestation or gestational):ti,ab,kw  #8: MeSH descriptor: [Pregnancy] explode all trees  #9: #7 or #8  #10: #6 and #9  **5.Scopus**  (TITLE-ABS-KEY(macrolide OR macrolides OR erythromycin OR clarithromycin OR azithromycin) AND TITLE-ABS-KEY (pregnant OR pregnancy OR pregnancies OR maternal OR prenatal OR gestation OR gestational)) AND (LIMIT-TO (DOCTYPE ,"ar") ) AND (LIMIT-TO (SRCTYPE , "j")) AND (LIMIT-TO (LANGUAGE , "English")) |
| --- |


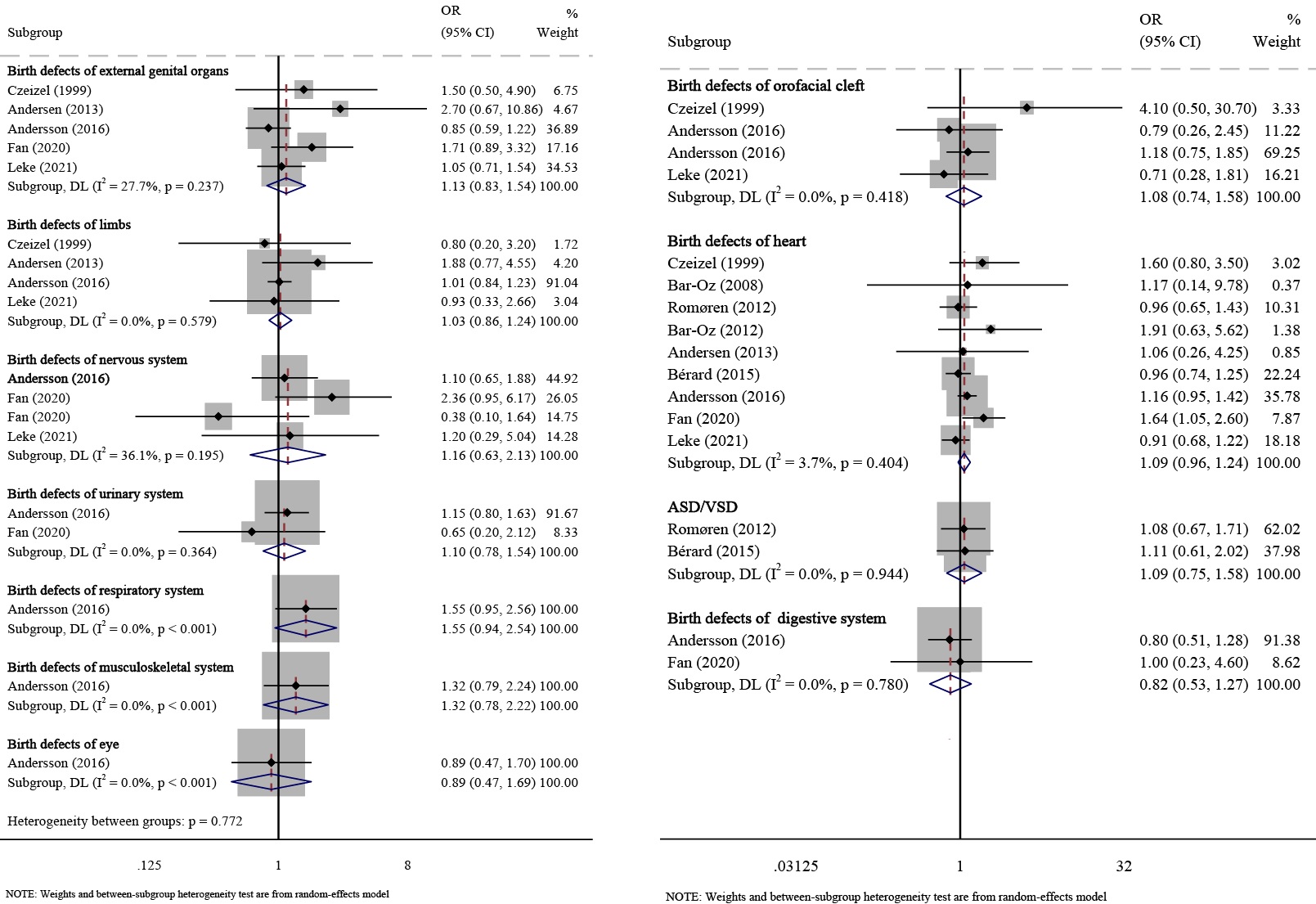


**Supplementary Figure 1: Subgroup analysis results for random effects model**
